# Evaluation of Rearing Parameters of a Self-Limiting Strain of the Mediterranean Fruit Fly, *Ceratitis capitata* (Diptera: Tephritidae)

**DOI:** 10.3390/insects11100663

**Published:** 2020-09-27

**Authors:** Rachid Elaini, Romisa Asadi, Neil Naish, Martha Koukidou, Mazih Ahmed

**Affiliations:** 1 Hassan II Institute of Agronomy and Veterinary Medicine, Complexe Horticole d’Agadir, Ait Melloul 80150, Morocco; ahmedmazih@gmail.com; 2 Omnium Agricole du Souss, Tassilla III, Agadir 80000, Morocco; 3 Oxitec Ltd., 71 Innovation Dr, Milton Park, Milton, Abingdon OX14 4RX, UK; neil.naish@oxitec.com (N.N.); martha.koukidou@gmail.com (M.K.)

**Keywords:** Mediterranean fruit fly, genetic-sexing, mass-rearing, insect pest management, sterile insect technique

## Abstract

**Simple Summary:**

The Mediterranean fruit fly (medfly), *Ceratitis capitata*, causes billions of dollars in losses to a wide variety of fruit and vegetable crops. Therefore, it needs to be effectively controlled. The use of chemical control is not sustainable because of its negative effects on the environment. As an alternative, the use of an area-wide sterile insect technique (SIT) has been shown to be a cost-effective and environmentally friendly option. This technique involves the mass rearing, irradiation for sterility, and release of males. Matings between wild females and sterile males result in no offspring production. The genetically sterile female specific medfly strain OX3864A has the potential to reduce the population of medfly through periodic male releases in a process similar to the common SIT. This study looked at ways of optimizing the mass rearing of the OX3864A strain. We found that the highest egg production is reached when mass-rearing cages are populated with pupal densities ranging from 14,000 to 18,000 per cage. In parallel, the optimal embryo densities for strain propagation and male-only pupal production are recommended. Using these densities, the quality of the insects meets the common standards.

**Abstract:**

The Mediterranean fruit fly (medfly), *Ceratitis capitata*, is a significant pest of stone and pome fruit that causes considerable economic losses worldwide. Current control is primarily based on insecticides, which are often mixed with protein baits. Oxitec has developed a self-limiting medfly strain (OX3864A) that demonstrates conditional female-specific mortality in the early life stages. Sustained release of OX3864A males offers medfly control, which should lead to substantial economic benefits in area-wide programmes. In the current study, the optimum quantities of mature and immature stages of the strain are assessed under semi-mass production. Moreover, the rearing and quality control limitations related to the production of this strain are provided. The data here demonstrate that the egg hatch rate can reach >85% under optimum rearing conditions. However, this depends on the number of pupae loaded in a cage and their ages. The suggested pupal density ranges between 14,000 and 18,000 pupae per cage to provide optimum egg production. In parallel, the embryo densities of 1.25–1.5 mL/kg larval Tet+ diet are recommended for strain propagation, while embryo densities of 1.25–2.0 mL/kg larval Tet− diet are suggested for male-only pupal production.

## 1. Introduction

The Mediterranean fruit fly, *Ceratitis capitata* (Wiedemann) (Diptera: Tephritidae) (hereafter, medfly), is an important pest of stone and pome fruit worldwide [1], due to its great capacity for dispersion and adaptability as well as its high rate of reproduction [2,3]. Because it adds to significant losses in fruit yield and quality [4], expensive quarantine measures that prevent or hinder agricultural exports from medfly-endemic countries have been established.

Insecticide sprays have traditionally been the principal tool for medfly control globally. Public concern regarding the health and environmental impacts of insecticide use, loss through de-registration of some insecticides, and the development of insecticide resistance [5,6,7,8,9] have driven the development of sustainable approaches to control this insect pest. Mass-trapping [10] and the sterile insect technique (SIT) [11] have been employed for medfly management. The sterile insect technique has enabled local eradication, prevention, and suppression of the medfly [11,12,13,14]. Examples of eradication successes of other species using SIT include the New World screwworm, *Cochliomyia hominivorax* (Coquerel), from North and Central America and from Libya [15]; the tsetse fly, *Glossina austeni* (Newsted), from Unguja Island in Zanzibar, Tanzania [16]; and the melon fly, *Bactrocera cucurbitae* (Coquillett), from Japan [17].

The SIT relies on the mass production of factory-reared insects and their subsequent sterilisation through irradiation. Sterile insects are released on a sustained basis in the environment to achieve an appropriate overflooding ratio. The sterile males mate with wild females, leading to no or few viable offspring being produced, causing the eventual reduction of the pest population. Higher overflooding ratios could result in local eradication [18]. The sterile insect technique implementation requires the development of a cost-effective mass-rearing system [19] that includes cost-efficient larval and adult diets [20,21,22,23].

Irradiation is known to induce dominant lethal genetic effects in the male germline, which render males unable to produce viable progeny, but irradiation also causes damage to somatic cells, resulting in reduced male competitiveness and higher operational costs [12,23,24,25,26,27,28,29,30]. The release of insects with ‘self-limiting’ genetic traits that induce early life-stage mortality to progeny has been demonstrated as an effective management tool for specific insects across both the agricultural and public health sectors [31,32,33,34,35]. The female-specific self-limiting approach allows for a male-only release cohort that has been previously shown to increase the effectiveness of mating-based insect pest management programmes [36]. Mating between self-limiting males and wild females produces no viable female offspring, thereby decreasing the wild population. The addition of an antidote to the larval rearing medium prevents the expression of the self-limiting gene, allowing for the normal propagation of the strain.

The medfly strain OX3864A, which was genetically engineered to carry a conditional female-specific self-limiting gene, has demonstrated full penetrance and benefits from an inherited genetic marker that can be quickly and accurately distinguished from its wild counterparts. The tetracycline transactivator (tTAV) accumulates in the absence of tetracycline, resulting in female death at the larvae and early pupal stages. The absence of tetracycline causes female lethality in early developmental stages, and only males survive to pupate. Provision of tetracycline to larval stages suppresses transgene lethality, allowing production of male and female pupae. The potential of the sustained release of OX3864A males to control wild-type medfly populations has been demonstrated previously [37].

A vital prerequisite for any mating-based insect control programme is the optimal rearing of the insect strain for propagation and, more importantly, the production of a male-only release cohort. This work evaluates the egg and adult densities that lead to optimum production and provides the rearing and quality control parameters associated with the strain.

## 2. Materials and Methods

### 2.1. Adult Colony Maintenance

The OX3864A strain was maintained at the insect-rearing facilities of the Omnium Agricole du Souss Macrobials Production Site, Chtouka, Morocco. All medfly life stages were maintained in a controlled environment (relative humidity (RH): 60–65%, 12:12 light:dark cycle), with adults reared at 25 ± 1 °C and larvae at 28 ± 1 °C.

Adults were maintained in a wooden frame cage with the following dimensions 77 cm × 7 cm × 72 cm (length × width × height). The two large sides of the cage were covered with an insect-proof mesh as an oviposition panel. Food was provided as a 1:4 mixture of enzymatic yeast hydrolysate (Biokar Diagnostics, A1202HA) and sugar. Water was provided and contained tetracycline (Sigma, T3383) at a concentration of 100 mg/L. The cages were suspended on metallic frames, with each frame holding approximately 11 cages.

### 2.2. Egg Production

Eggs were oviposited through a fine mesh on the side wall of each cage and collected in troughs of water beneath the cages during a 24-h period. The egg–water solution was passed through a fine sieve, and the retained eggs were washed off with a water bottle into a volumetric cylinder. Following volume measurement, eggs were transferred onto a damp filter paper inside a 90-mm Petri dish and kept there for 48 h.

To evaluate optimal egg production, each cage was populated with different volumes of pupae at an assumed 1:1 male to female ratio. Tested densities ranged between 2000 and 24,000 pupae per cage (at intervals of 2000), and each density was replicated seven times. Eggs were collected daily between days 5 and 24 post adult emergence, and their numbers were estimated volumetrically, accounting for 22,100 eggs per mL (unpublished data/previous study). The total egg production per cage was calculated at the end of the collection period. The pre-oviposition period (time between emergence and first egg collection) and the period between the first and last egg collections were also recorded for each cage.

### 2.3. Pupal Production

Eggs were seeded onto one kilogram of larval rearing medium, as described by Tanaka et al. [38], using plastic trays (25.0 cm × 13.0 cm × 5.0 cm) (length × width × height) with added tetracycline at a concentration of 100 mL/L for strain maintenance (hereafter, Tet+), or without additives for male-only cohorts (male-only releases) (hereafter, Tet−).

Third-instar larvae crawled up the side of the plastic container containing the larval rearing medium and pupated directly onto a thin layer of sterilised sand. The sand was sieved daily for 5 days to allow for synchronous adult emergence in each collected batch of pupae.

To evaluate the optimal level of pupal production, different egg volumes (0.25–3.00 mL at 0.25 intervals) were seeded per 1 kg of rearing medium, as described above. Six replicates were tested for each egg volume. This experiment was performed for pupal production (males and females), then for male-only production.

### 2.4. Quality Control Parameters

#### 2.4.1. Egg Production

During each egg production experiment, 100 egg samples were taken at 24-h intervals to measure the egg hatch rate. Each sample was placed on a damp filter paper within a 90-mm Petri dish (to avoid desiccation) and allowed to develop to the larval stage. The number of unhatched or damaged eggs was recorded. The pupal recovery (%) was calculated by dividing the number of pupae by the initial number of eggs placed in each cage.

#### 2.4.2. Pupal Production

Daily pupal production was measured volumetrically. The volume (mL), weight (g), and developmental time from egg seeding to the appearance of the first pupae were measured for each pupal cohort. The total number of pupae obtained from each treatment was determined by dividing the total weight of the pupae in each collection by the average weight of one pupa (mean of 100-pupae sample from each pupal collection) [39].

### 2.5. Statistical Analysis

Comparisons of egg production between cages with different pupal densities and pupal production between different egg densities were performed using one-way analysis of variance (ANOVA) with a post-hoc Tukey Hogan Development Survey (HSD) test. For this study, p-values of ≤0.05 were considered statistically significant. The regression analysis was performed to determine how egg and pupal production were affected by pupal and egg densities respectively. All statistical analyses were performed using Minitab 16 software (Minitab, Inc., State College, PA, USA).

## 3. Results

### 3.1. Egg Production

**Figure 1:**
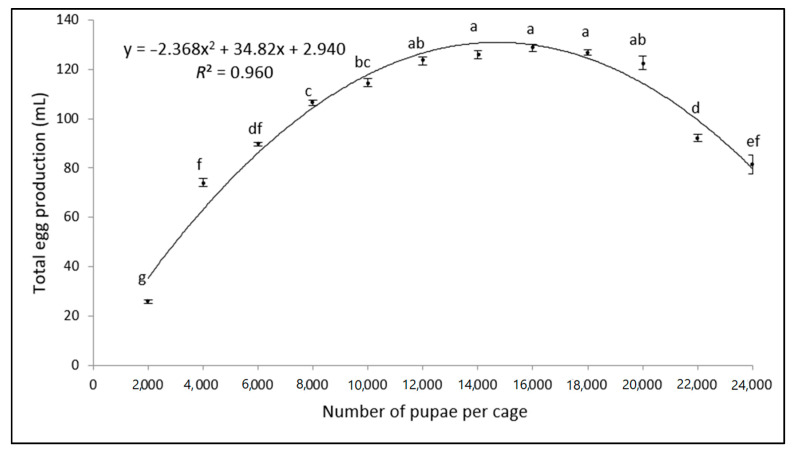
Comparative analysis of the total volume of eggs (mL) obtained from cages populated with varying amounts of OX3864A pupae (mean ± *SD*; *n* = 7). Bars superscripted with different letters are significantly different according to the ANOVA and Tukey’s HSD tests (*p* > 0.05).

Significant differences were observed in total egg production across the tested density range (*F* = 264.65; d.f. = 11; *p* < 0.001) (Figure 1). For densities between 2000 and 18,000 pupae per cage, a relationship between the pupal density and total egg production was observed, while higher pupal densities (>18,000 pupae per cage) negatively affected the total egg production. The coefficient of determination (*R*^2^) between the pupal volume and total egg production was 96% (y = −2.36x^2^ + 34.82x + 2.94; *R*^2^ = 0.9602), indicating that the curve fitted the data well. These data show that the peak egg production per cage can be reached with pupal densities ranging between 14,000 and 18,000 pupae per cage, with pupal densities outside of this range yielding significantly fewer eggs.

**Figure 2:**
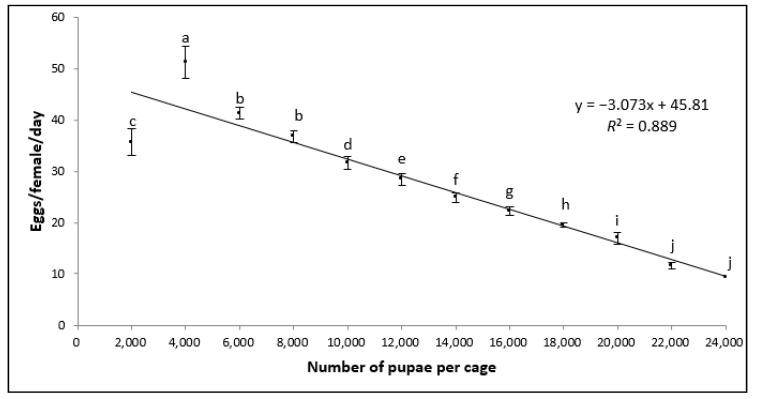
Estimated number of eggs produced per female per day within cages populated with different pupal densities (mean ± *SD*; *n* = 7). Bars superscripted with different letters are significantly different according to the ANOVA and Tukey’s HSD tests (*p* > 0.05).

Regarding the daily oviposition rate per female per cage (Figure 2), statistically significant differences were observed among pupal densities (*F* = 580.32; d.f. = 11; *p* < 0.001). A negative relationship was observed between the number of females per cage and the number of eggs produced per female. The coefficient of determination (*R*^2^) for the total oviposition rate per female between pupal densities was 89%.

No statistically significant differences were observed among pupal densities for other egg production parameters such as (i) the time between first adult emergence and sexual maturation (as determined by the start of oviposition) (5.01 ± 0.28 days; *F* = 0.76; d.f. = 11; *p* = 0.674); (ii) the period during which oviposition was observed (16 ± 0.43 days; *F* = 0.76; d.f. = 11; *p* = 0.677), and (iii) the mean egg hatch rate (88.7 ± 1.37%; *F* = 1.55; d.f. = 11; *p* = 0.133). High-quality eggs (determined by hatch rates >85%) were produced for an average of 2 weeks until approximately 3 weeks post adult emergence.

**Figure 3:**
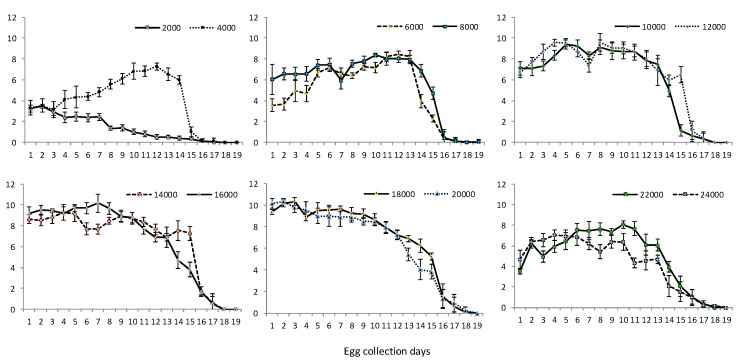
Average daily fecundity of OX3864A females in cages populated with variable pupal densities (mean ± *SD*; *n* = 7). Egg collection commenced at day 5 post adult emergence for all cages.

The daily egg volume (mL), and, therefore, egg production, for each cage treatment are shown in Figure 3. Generally, for all cage pupal density treatments, eggs were not produced beyond 23 days post emergence; however, a decline in egg production was observed in most cage treatments by 20 days post emergence. Egg production peaked on days 10–13 post emergence for cages with lower pupal densities (4000–12,000 pupae/cage) (Figure 3), except for the 2000 pupae density (peak production: days 6 and 7 post emergence). For higher pupal densities (14,000–20,000 pupae per cage), peak egg production was observed on days 7–10 post emergence (Figure 3). Increased egg volumes were obtained at days 10–11 post emergence (Figure 3) for the highest pupal densities (22,000 and 24,000 pupae per cage).

### 3.2. Pupal Production

**Figure 4:**
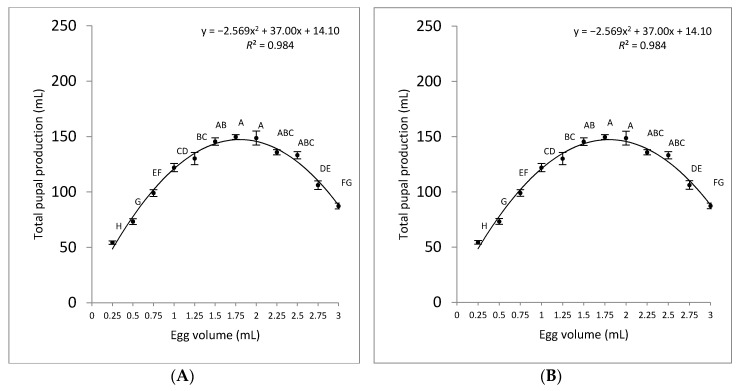
Comparison of pupal production (mL) for different volumes of OX3864A eggs seeded into 1 kg of larval rearing medium under permissive (Tet+) (**A**) and restrictive (Tet−) (**B**) conditions. The mean volume of pupae produced is shown for each initial egg density (mean ± *SE*; *n* = 6). Mean values labelled with the same letter are not significantly different according to ANOVA and Tukey’s HSD tests (*p* > 0.05).

The larval development time was significantly higher in the presence of tetracycline (Tet+; *F* = 74.84; d.f. = 11; *p* < 0.001) than in its absence (Tet−; *F* = 93.00; d.f. = 11; *p* < 0.001). This was expected, as the female-specific self-limiting trait affects females at the early stages, largely at the pre-pupal developmental stage. A direct link between pupal production (mL) and egg density was observed up to a density of 1.5 mL eggs/kg for both Tet+ and Tet− treatments (Figure 4; Panels A and B, respectively). Higher egg densities resulted in the production of fewer pupae. The coefficients of determination (*R*^2^) between pupal production and egg densities per kg were 92% and 98% for Tet+ and Tet−, respectively. The data indicate that the egg density of 1.25 mL/kg on Tet+ rearing medium resulted in the highest number of pupae, while egg densities of 1.5–2 mL/kg on Tet− rearing medium yielded the highest quantities of male-only pupae.

Significant differences in the total pupal production and pupal weight were observed for different volumes of eggs seeded into 1 kg of (Tet+) rearing medium (*F* = 39.66; d.f. = 11; *p* < 0.001) (Table 1). The highest pupal production values resulted from an egg density of 1.25 mL/kg larval rearing medium. However, the heaviest pupae originated from egg densities between 0.25 mL/kg and 0.75 mL/kg (average weight = 9 mg per pupa). Significant differences in the larval development time were also observed (*F* = 4.53; d.f. = 11; *p* < 0.001), with higher egg densities leading to longer times (12 days) for pupal recovery. In contrast, the egg-to-pupa recovery (*F* = 122.05; d.f. = 11; *p* < 0.001) and pupal weight per 100 pupae (*F* = 13.53; d.f. = 11; *p* < 0.001) were negatively affected by higher egg densities. However, no significant differences in the duration of pupal collection (4 days for all egg densities) were found (*F* = 1; d.f. = 11; *p* = 0.453). Male-only pupal production was highest at the density of 2 mL eggs per 1 kg of Tet− rearing medium (Table 2). No significant differences were observed for the time required for complete larval development (10 days; *F* = 0.9; d.f. = 11; *p* = 0.574) or the duration of pupal collection (4 days; *F* = 1.45; d.f. = 11; *p* = 0.174) between egg densities. Higher egg densities negatively affected the egg-to-pupae recovery rate (*F* = 1.45; d.f. = 11; *p* < 0.001) and the mean pupal weight per 100 pupae (*F* = 13.53; d.f. = 11; *p* < 0.001), with the highest pupal recovery rate of 59.69 ± 1.99 being obtained at a density of 0.25 mL/kg of larval rearing medium. In contrast, the lowest pupal recovery rates (10.20 ± 0.56 and 8.00 ± 0.2) were recorded at egg densities of 2.75 mL/kg and 3.0 mL/kg of Tet− rearing medium, respectively.

Regarding the egg density, a sample of 100 pupae was tested for adult emergence on Tet+ and Tet− rearing mediums. No significant differences were observed for male (*F* = 0.35; d.f. = 11; *p* = 0.969), female (*F* = 1.2; d.f. = 11; *p* = 0.305), or total adult (*F* = 1,44; d.f. = 11; *p* = 0.178) emergence rates, as well as pupal mortality rates (9.58 ± 0.508%; *F* = 1.23; d.f. = 11; *p* = 0.271) with the Tet+ rearing medium. Male (*F* = 0.79; d.f. =1 1; *p* = 0.648) and total adult (*F* = 0.79; d.f. = 11; *p* = 0.648) emergence rates, as well as pupal mortality rates (7.88 ± 0.797%; F = 0.72; d.f. = 11; *p* = 0.713) were also similar with the Tet− rearing medium. For all egg densities on the Tet+ and Tet− rearing mediums, the first day of pupal collection yielded the highest number of pupal volumes, while the pupae yield from the fourth day was very low (less than 10% of the total production).

## 4. Discussion

The sterile insect technique programmes have been successful worldwide at locally suppressing or eradicating pest populations of medfly [40]. However, their cost effectiveness might be compromised by the irradiation employed to sterilise males and the resulting decrease in mating competitiveness. OX3864A does not use irradiation, and therefore, in this study, it was hypothesized to be a more cost-effective alternative to pest population suppression, provided mass-rearing of the strain shows comparable standards to SIT strains.

Under the conditions tested in this study, and at a cage density of 18,000 pupae, the mean egg production for OX3864A was double the reported egg production for the SIT strain VIENNA-8 *tsl* (temperature sensitive lethal), a genetic sexing strain tested by Neto et al. [21]. Furthermore, Rempoulakis et al. (2016) reported lower egg production for the three *tsl* strains (VIENNA-8, VIENNA-8.Sr^2^, and VIENNA 8-1260). The current data also showed that egg hatching rates across all experimental treatments were 27–48% higher than those reported for the VIENNA-8 *tsl* strain by Neto et al. [21] and Rempoulakis et al. [41]. Caceres et al. (2002) directly linked the main cost differences in rearing a *tsl*-based genetic sexing strain and a wild-type strain to their respective egg production efficiencies. Taken together, the higher egg yields, better egg hatch rates, and high stability of the OX3864A Oxitec medfly strain translated into a cost reduction of 59% to produce 100 million males per week, when compared to the *tsl* (Table 3), thus representing a significant cost saving in a mass-rearing setting. These calculations were based on Caceres et al.’s (2002) description of the relative efficiencies of a *tsl* SIT genetic sexing strain, including staffing requirements, consumables, equipment, and facility rental.

To obtain maximum efficiency, egg collections should be timed to ensure a high number of eggs are fertilized in the shortest possible time. Considering factors such as the sexual maturation of female medflies (~day 3 post emergence; [28,42,43,44]), mating period (~days 3–5 post emergence; [45]), peak egg production, and high egg hatch rates (>85%), the recommended egg collection period for all cage densities is between days 5–19 post adult emergence. Egg collections performed after 9 days were associated with increased female mortality within the cages and decreased female fecundity, suggesting reduced cost efficiency for a mass-rearing system. This optimal two-week egg collection period is comparable to that identified with the VIENNA-8 strain (Hamden et al., 2013) and ensures a steady and uninterrupted supply of sterile OX3864A males during a suppression programme.

Previously, in cage studies, we showed the capacity of the self-limiting OX3864A strain to suppress wild-type medfly populations [37]. Accompanied by the present study, these studies investigated the mass-rearing parameters related to strain propagation and production of a male-only cohort for field release in a mating-based operational programme. These data demonstrate the potential of the OX3864A strain to be mass-reared successfully and, potentially, more cost-effectively than previous SIT strains. Further in-country analyses will be required before the self-limiting strain OX3864A becomes part of an operational programme for medfly control.

## 5. Conclusions

The sterile insect technique, when used for the control of the Mediterranean fruit fly (medfly), *Ceratitis capitata* (Wiedemann) (Diptera: Tephritidae), relies on the release of sterile flies of only the male sex. Its success depends on a consistent mass rearing of quality insects. Their production is directly related to the availability, suitability, and cost of the diet ingredients used. This study allowed us to implement and optimize, for the first time, the mass rearing of the genetically engineered OX3864A medfly strain.

Our results demonstrate that adult and immature stage densities significantly affect the mass rearing and quality of these flies. In fact, the peak egg production per cage can be reached with pupal densities ranging between 14,000 and 18,000 pupae per cage. The highest pupal production values resulted from an egg density of 1.25 mL/kg larval Tet+ diet, while egg densities of 1.5–2 mL/kg on Tet− rearing medium yielded the highest quantities of male-only pupae. Quality parameters such as the egg hatch rate, the pupal weight, and the emergence rate meet recommended standards. These data demonstrate the potential of the OX3864A strain to be mass-reared successfully and, potentially, more cost-effectively than previous SIT strains. Further in-country analyses will be required before the self-limiting strain OX3864A becomes part of an operational programme for medfly control.

## Figures and Tables

**Table 1:** Comparative pupal recovery rates (%) and quality control parameters for different volumes of OX3864A eggs seeded into 1 kg of rearing medium under permissive conditions (Tet+) (mean ± *SE*; *n* = 6). Data followed by the same letter in the same column do not differ significantly according to ANOVA and Tukey’s HSD tests (*p* > 0.05).

Volume of Eggs (mL)	Total Pupal Weight (g)	Weight per 100 Pupae (g)	Number of Pupae	Pupal Recovery (%)
0.25	39.22 ± 1.13 ^g^	0.90 ± 0.00 ^a^	4358 ± 125 ^g^	69.97 ± 4.78 ^a^
0.5	81.06 ± 1.92 ^de^	0.90 ± 0.00 ^a^	9006 ± 214 ^de^	77.36 ± 5.30 ^a^
0.75	98.12 ± 2.71 ^abcd^	0.90 ± 0.00 ^a^	10,902 ± 301 ^cd^	59.81 ± 1.15 ^b^
1	105.35 ± 3.44 ^abc^	0.83 ± 0.02 ^ab^	12,662 ± 425 ^bc^	51.72 ± 2.89 ^bc^
1.25	116.76 ± 9.47 ^a^	0.72 ± 0.02 ^c^	16,334 ± 1402 ^a^	59.65 ± 5.73 ^b^
1.5	118.80 ± 10.80 ^a^	0.75 ± 0.02 ^bc^	15,787 ± 1294 ^ab^	45.43 ± 3.84 ^cd^
1.75	117.58 ± 4.41 ^a^	0.75 ± 0.02 ^bc^	15,743 ± 770 ^ab^	40.66 ± 1.95 ^de^
2	113.12 ± 6.02 ^ab^	0.72 ± 0.02 ^c^	15,831 ± 944 ^ab^	36.42 ± 4.16 ^e^
2.25	92.42 ± 1.51 ^bcd^	0.80 ± 0.04 ^abc^	9315 ± 199 ^de^	18.73 ± 0.40 ^f^
2.5	83.50 ± 1.58 ^cde^	0.78 ± 0.03 ^bc^	8784 ± 171 ^def^	15.90 ± 1.31 ^f^
2.75	64.27 ± 0.75 ^ef^	0.82 ± 0.03 ^abc^	6762 ± 68 ^efg^	11.13 ± 1.11 ^f^
3	52.57 ± 1.64 ^fg^	0.75 ± 0.02 ^bc^	5717 ± 133 ^fg^	8.62 ± 0.20 ^f^

**Table 2:** Comparative pupal recovery rates (%) and quality control parameters for different volumes of OX3864A eggs seeded into 1 kg of rearing medium under restrictive conditions (Tet−) (mean ± *SE*; *n* = 6). Data followed by the same letter in the same column do not differ significantly according to ANOVA and Turkey’s HSD tests (*p* > 0.05).

Volume of Eggs (mL)	Total Pupal Weight (g)	Weight per 100 Pupae (g)	Number of Pupae	Pupal Recovery (%)
0.25	29.68 ± 0.99 ^e^	0.90 ± 0.00 ^a^	3298 ± 110 ^g^	59.69 ± 1.99 ^a^
0.5	40.70 ± 1.42 ^de^	0.90 ± 0.00 ^a^	4522 ± 158 ^fg^	40.93 ± 1.43 ^b^
0.75	60.47 ± 4.06 ^bc^	0.90 ± 0.00 ^a^	6719 ± 451 ^de^	40.53 ± 2.72 ^bc^
1	69.07 ± 2.71 ^ab^	0.85 ± 0.02 ^ab^	8151 ± 378 ^cd^	36.88 ± 1.71 ^bcd^
1.25	72.22 ± 4.11 ^ab^	0.77 ± 0.02 ^bc^	9399 ± 374 ^bc^	34.02 ± 1.35 ^cde^
1.5	81.93 ± 2.06 ^a^	0.77 ± 0.02 ^bc^	10,725 ± 376 ^ab^	32.35 ± 1.13 ^def^
1.75	82.87 ± 2.12 ^a^	0.72 ± 0.02 ^c^	11,585 ± 356 ^a^	29.96 ± 0.92 ^ef^
2	84.13 ± 3.51 ^a^	0.70 ± 0.00 ^c^	12,018 ± 502 ^a^	27.19 ± 1.14 ^f^
2.25	80.02 ± 1.41 ^a^	0.78 ± 0.03 ^bc^	8064 ± 188 ^cd^	16.22 ± 0.38 ^g^
2.5	85.55 ± 8.30 ^a^	0.78 ± 0.03 ^bc^	8576 ± 71 ^c^	15.52 ± 0.13 ^g^
2.75	62.13 ± 2.47 ^bc^	0.75 ± 0.02 ^c^	6200 ± 343 ^e^	10.20 ± 0.56 ^gh^
3	51.32 ± 1.47 ^cd^	0.77 ± 0.02 ^bc^	5303 ± 136 ^ef^	8.00 ± 0.21 ^h^

**Table 3:** Comparison of rearing requirements and rearing costs between *tsl* and OX3864A to produce 100 million medfly males per week. (Caceres et al., 2002, personal communication with Oxitec Ltd. modelling team for OX3864A cost analysis). *tsl*: temperature sensitive lethal.

Criteria	*tsl*	OX3864A
Colony size (millions of adults)	22	12
Colony replacement (millions of pupae)	60	16
No. of cages for male only production	165	50
Egging room (m^2^); male only	437	133
No. of cages for colony maintenance	24	4
Total no. of cages	189	54
Egging room (m^2^); colony	78	11
Adult diet (sugar) (kg/day)	24	9
Adult diet (yeast hydrolysate) (kg/day)	8	3
Workers (colony + filter)	9	3
Total cost per million males	$510	$207
Factory and equipment	$124	$119
Consumables	$251	$33.80
Labour	$111	$44
Space	$23	$9
